# Adaptation of the Online Problem Gambling Behavior Index: Associations with Emotional Reactivity and Psychological Distress

**DOI:** 10.1007/s10899-025-10407-w

**Published:** 2025-09-01

**Authors:** Ayşen Kovan, Murat Yıldırım

**Affiliations:** 1 Eskişehir, Turkey; 2 Department of Psychology, Agri Ibrahim Cecen University, Ağrı, Turkey; 3 Psychology Research Center, Khazar University, Baku, Azerbaijan; 4 Department of Psychology, Faculty of Science and Letters, Agri Ibrahim Cecen University, Fırat Mahallesi Yeni Üniversite Caddesi No: 2 AE/1, Merkez, Ağrı, 04100 Turkey

**Keywords:** Online gambling, Scale adaptation, Emotion dysregulation, Psychological distress, Structural equation modeling

## Abstract

Problematic online gambling has emerged as a growing behavioral health concern, fueled by the accessibility, anonymity, and immediacy of digital gambling environments. This two-phase study aimed to adapt the “Online Problem Gambling Behavior Index (OPGBI)” for Turkish-speaking populations and to examine the affective mechanisms underlying problematic gambling behavior through the lens of the Emotion Dysregulation Model. In Study-I (*N* = 674; 62.6% male; M_age_ = 35.23), confirmatory factor analysis supported a three-factor structure: gambling behavior, limit-setting, and communication with operators, demonstrating strong internal consistency, good model fit, and gender-based measurement invariance. In Study-II (*N* = 813; 65% male; M_age_ = 34.76), structural equation modeling tested a mediation model in which emotional reactivity predicted problematic gambling behavior both directly and indirectly via psychological distress (depression, anxiety, and stress). Results demonstrated robust direct effects of emotional reactivity across all OPGBI dimensions, with stress emerging as the most consistent mediator. Bootstrapped analyses confirmed the significance of indirect pathways. Collectively, the findings validate the Turkish version of the OPGBI as a psychometrically sound, culturally responsive instrument and underscore the central role of emotional vulnerability in the development of online gambling problems.

## Introduction

In the digital age, problem gambling has evolved into a challenging public health issue, increasingly shaped by technological innovation and the ubiquity of online platforms. Traditionally confined to physical venues, gambling has become more accessible, instantaneous, and private through the rise of internet-based gambling services, including mobile apps, social media-integrated games, and virtual casinos (Gainsbury et al., 2015). The combination of 24/7 availability, anonymity, and immersive design features has significantly increased the potential for gambling-related harm, particularly among vulnerable individuals. According to the World Health Organization (WHO, 2024), behavioral addictions such as gambling disorder are associated with adverse psychological, financial, and social outcomes, often co-occurring with anxiety, depression, and emotional dysregulation. The American Psychiatric Association (APA, 2013) recognizes gambling disorder as a behavioral addiction in the Diagnostic and Statistical Manual of Mental Disorders (DSM, 5th ed.), further emphasizing the clinical relevance of this phenomenon. In Türkiye, recent studies have reported a worrying upward trend in online gambling activity, especially among young adults, with higher rates of problem gambling symptoms linked to increased internet use and emotional distress (Altıntaş et al., 2024; Kovan, 2025).

Moreover, in Türkiye, all forms of land-based casino gambling have been banned since 1998, and online gambling remains officially prohibited except for government-sanctioned sports betting platforms. Despite these legal restrictions, unregulated digital gambling sites have proliferated, especially among young adults and urban populations. This legal ambiguity creates a socio-political environment in which gambling behavior is often hidden, stigmatized, and difficult to measure accurately. Recent research suggests rising levels of gambling-related harm and an increasing demand for treatment among individuals using unauthorized platforms (Erkin, 2022; Kovan, 2024). Consequently, research in this domain faces unique ethical and methodological challenges, including underreporting and mistrust, further reinforcing the need for culturally adapted, context-sensitive measurement tools.

Problematic online gambling is not only seen as a behavioral addiction but has also emerged as a significant mental health concern, particularly because of its strong associations with emotional dysregulation, impulsivity, and maladaptive coping strategies. Especially as internet-based gambling platforms allow for constant and discreet access, individuals are more likely to use gambling as a mechanism to regulate negative emotions or escape psychological discomfort (Alaba-Ekpo et al., 2024). Most of the studies in the literature have indicated that those who engage in problematic online gambling often exhibit poor emotional regulation skills and heightened impulsivity, both of which are established risk factors for the onset and maintenance of addictive behaviors (Estevez et al., 2015; Mestre-Bach et al., 2023). The cognitive-behavioral model of gambling posits that distorted beliefs, such as the illusion of control and the gambler’s fallacy, coupled with emotion-driven urges, reinforce gambling as a dysfunctional coping mechanism (Pfund et al., 2023). Therefore, individuals may become trapped in a cycle where emotional distress fuels gambling behavior, which in turn exacerbates psychological symptoms.

Mounting empirical evidence supports the link between gambling and a broad spectrum of psychological distress symptoms, including depression, anxiety, and stress. Several large-scale studies and meta-analyses have reported significantly higher rates of depressive symptoms among individuals exhibiting problem gambling behaviors (Gooding & Tarrier, 2009; Quigley et al., 2015). Similarly, anxiety has been consistently identified as both a predictor and outcome of gambling problems, suggesting a bidirectional relationship (Afifi et al., 2016; Parhami et al., 2014). Furthermore, stress has been shown to contribute to increased gambling frequency and intensity, particularly during emotionally taxing life events or periods of uncertainty (Sachdeva et al., 2022). Thus, in online contexts, the immediacy and anonymity of gambling platforms may amplify these risks, making it easier for individuals to engage compulsively without social or environmental restraints (Gainsbury, 2015; Hing et al., 2022). Besides these, studies also demonstrate comorbidity between gambling disorder and other psychological issues such as substance use, ADHD, and personality disorders, highlighting the multidimensional clinical profile of affected individuals (Rash et al., 2016; Sharma & Weinstein, 2025). Taken together, these findings emphasize that online gambling is not merely a financial or recreational issue but a profoundly embedded psychological problem that warrants targeted clinical attention and culturally relevant assessment tools.

A theoretically grounded explanation for the emergence of maladaptive behaviors such as problematic online gambling can be found in the Emotion Dysregulation Model (EDM) developed by Weiss et al. (2012). This model builds upon the foundational work of Gratz and Roemer (2004), who conceptualized emotion dysregulation as a multidimensional construct involving difficulties in understanding, accepting, and modulating emotional responses, particularly during heightened emotional states. According to this framework, individuals with elevated emotional reactivity, those who experience emotions more intensely and rapidly, are at greater risk of engaging in impulsive and avoidance-oriented behaviors when confronted with distress. Online gambling, with its immediate access and emotionally immersive nature, may serve as a maladaptive strategy to escape or manage overwhelming affective experiences. Furthermore, persistent difficulties in regulating emotional states are closely linked to elevated levels of psychological distress, including symptoms of depression, anxiety, and stress (Gratz & Roemer, 2004). The EDM thus can present a coherent lens through which the associations between emotional vulnerability, psychological disturbance, and problematic online gambling behavior can be understood. In particular, it can provide a suitable theoretical foundation for examining how individual differences in emotional reactivity and regulation contribute to the escalation and maintenance of gambling-related problems in digital environments.

Although the proliferation of online gambling has introduced new dimensions of risk, empirical efforts to assess this behavior have often been constrained by measurement tools that inadequately capture its digital-specific features, particularly in non-Western populations. At this point, the Online Problem Gambling Behavior Index (OPGBI), developed by Auer et al. (2024), represents a meaningful shift in the assessment of gambling pathology by moving beyond consequence-based frameworks toward the behavioral mechanisms that underpin problematic online gambling. Developed using empirical behavioral data rather than retrospective self-report of harms, the OPGBI reflects contemporary trends in digital gambling engagement, including patterns of persistence, preoccupation, and diminished control. Psychometrically, the scale has demonstrated a three-factor structure, “Gambling Behavior, Limit-Setting, and Communication with Operators”, each capturing distinct behavioral aspects of problematic online gambling. It also showed high internal consistency (α = 0.82) and convergent validity with established indices of gambling severity, indicating its soundness as a measurement tool in its original context. However, despite its relevance, no culturally or linguistically adapted version of the OPGBI currently exists for Turkish-speaking populations a significant omission given the increasing prevalence of online gambling and its psychosocial ramifications in this region. The adaptation of psychometrically validated tools across cultural settings is not a superficial exercise in translation but a critical endeavor that ensures conceptual equivalence, cultural sensitivity, and contextual relevance (Beaton et al., 2000; Van de Vijver & Tanzer, 2004). Without such adaptation, research efforts remain epistemologically narrow and clinical interventions risk misalignment with culturally specific expressions of gambling behavior. In this light, adapting the OPGBI is both a scientific and ethical necessity and may allow for a detailed exploration of online gambling behaviors in underrepresented populations and contribute to the global comparability of research findings.

### Associations with Emotional Reactivity and Psychological Distress

Emotional reactivity, the tendency to experience intense and persistent emotional responses to stimuli, has been identified as an important vulnerability factor in the development of various maladaptive behaviors, including those related to addiction (Miela et al., 2018). Individuals high in emotional reactivity often report being easily overwhelmed by emotional stimuli and experiencing difficulty returning to baseline states (Charles, 2010). Within the context of the EDM (Weiss et al., 2012), such individuals are more likely to engage in externally focused strategies to escape or suppress emotional discomfort. This is particularly relevant in digital environments where gambling platforms provide immediate, immersive distraction and temporary relief from aversive internal states (Hodgins & Holub, 2015). Emotional reactivity thus acts as a key dispositional factor that may initiate and maintain engagement in online gambling as a form of affect regulation (Cholewich & Bennett, 2025). Importantly, high emotional reactivity is often accompanied by lower thresholds for distress tolerance and reduced access to adaptive regulation strategies and patterns that can escalate gambling behavior into a clinically significant problem (Williams et al., 2012).

Parallel to this, psychological distress encompassing symptoms of depression, anxiety, and stress has consistently been linked to increased gambling severity and persistence (Afifi et al., 2016; Gooding & Tarrier, 2009; Parhami et al., 2014; Sachdeva et al., 2022; Quigley et al., 2015). Studies show that individuals experiencing high levels of psychological distress are more likely to use gambling as a coping mechanism, particularly when other emotion regulation resources are insufficient (Pfund et al., 2023; Velotti et al., 2021; Williams et al., 2012). Longitudinal studies indicate that psychological distress predicts future gambling problems and mediates the relationship between emotional vulnerability and gambling-related harm (Devos et al., 2020; Hartmann & Blaszczynski, 2018). Moreover, digital gambling platforms, by their constant availability and anonymity, may intensify this dynamic by lowering barriers to impulsive use during emotional crises (Gainsbury et al., 2015). The inclusion of emotional reactivity and psychological distress as focal psychological correlates in this study not only demonstrates compatibility with existing theoretical frameworks but also allows for the validation of the adapted OPGBI. Their inclusion may help to assess whether the scale can meaningfully discriminate between normative and clinically concerning gambling patterns, thereby potentially increasing its utility for both research and clinical screening in the target population (Estevez et al., 2015; Hodgins et al., 2011; Parhami et al., 2014).

Although online gambling has become a global behavioral health concern, research in this domain remains disproportionately concentrated in Western populations, with culturally and linguistically validated instruments for non-Western contexts notably lacking. This gap is particularly pronounced in the Turkish context (Kovan, 2025), where rising online gambling engagement has not been met with methodological tools capable of capturing the behavioral and psychological aspects of this phenomenon. Moreover, existing literature rarely integrates theoretical frameworks that account for the emotional processes underpinning gambling behavior. Studies that incorporate models such as the EDM remain sparse, and those that do often overlook the combined utility of psychometric validation and theory-driven analysis. The present study addresses these important gaps through a two-pronged approach: (i), by culturally adapting and validating the OPGBI for use in Turkish-speaking populations; and (ii), by empirically examining the relationships between emotional reactivity, psychological distress, and online gambling behavior within this framework. By uniting culturally grounded psychometric methodology with a theoretically anchored exploration of psychological mechanisms, this study is thought to provide a comprehensive and contextually sensitive contribution to the literature. Through this, it cannot only improve the measurement precision of online gambling in underrepresented populations but also enhance conceptual understanding of how emotional vulnerabilities manifest in digitally mediated risk behaviors.

### The Present Study

In response to the urgent need for culturally sensitive tools and theory-driven inquiry in the domain of online gambling, the present study adopts a dual-phase design aimed at both methodological and conceptual advancement. The first aim is to adapt and validate the OPGBI for Turkish-speaking populations, addressing the current absence of psychometrically robust instruments tailored to this cultural context. The second aim is to examine the emotional and psychological correlates of problematic online gambling through the lens of the EDM, thereby integrating the individual difference theory into the study of gambling behavior an approach still underrepresented in the literature. Based on these explanations, Study-I focuses on the cultural adaptation and psychometric evaluation of the OPGBI, including confirmatory factor analysis (CFA) to assess structural validity, reliability assessments, and measurement invariance testing across gender. Followed by, Study-II employs a mediation framework using Hayes’ Process Macro to test theoretically grounded hypotheses. These hypotheses are addressed as follows:

#### H_1_

Emotional reactivity will be positively associated with the three dimensions of problematic online gambling behavior: gambling behavior, limit-setting, and communication with operators. This hypothesis reflects the core premise of the EDM (Weiss et al., 2012), which suggests that individuals with heightened emotional sensitivity and difficulty modulating affect are more likely to engage in maladaptive behaviors (such as persistent online gambling) to regulate or escape from distressing emotional states.

#### H_2a_

Depression will mediate the relationship between emotional reactivity and each of the three dimensions of problematic online gambling behavior.

#### H_2b_

H_2b_. Anxiety will mediate the relationship between emotional reactivity and each of the three dimensions of problematic online gambling behavior.

#### H_2c_

H_2c_. Stress will mediate the relationship between emotional reactivity and each of the three dimensions of problematic online gambling behavior. These mediation hypotheses are grounded in the EDM and supported by empirical evidence indicating that psychological distress manifesting as depression, anxiety, or stress emerges from heightened emotional reactivity and, in turn, increases the likelihood of engaging in emotionally avoidant behaviors such as online gambling.

## Method

### Study-I

Study-I aimed to translate, culturally adapt, and psychometrically validate the OPGBI for use in the Turkish context. Given the rapid rise of online gambling and its associated harms, the availability of valid and reliable measurement tools is essential for both research and clinical assessment. Although the original OPGBI was developed as a behavior-focused alternative to consequence-based gambling scales, no Turkish adaptation has previously been conducted and similar measurement tools are not available. Following internationally recognized guidelines for cross-cultural scale adaptation (Beaton et al., 2000; Van de Vijver & Tanzer, 2004), the current study evaluated the scale’s construct validity, factor structure, internal consistency, and measurement invariance across gender. CFA was used to test the three-factor model of the original instrument, comprising Gambling Behavior, Limit-Setting, and Communication with Operators. Reliability was assessed through multiple indices, and potential method bias was examined to ensure psychometric robustness.

#### Participants and Procedure

Data were collected through an online, self-administered survey hosted on a digital platform. Participants were recruited through targeted calls for participation posted on online platforms, social media groups, and digital communities related to sports betting, gaming, and general entertainment platforms where gambling behavior is commonly discussed (e.g., Telegram betting groups and Twitter/X gambling-related community pages). Eligibility criteria required participants to be < 18 years of age and self-report current or previous engagement in online gambling activities. To ensure relevance to the study focus, participants were asked a screening question during the consent process: “Have you participated in any form of online gambling (e.g., online betting, virtual casinos, or games of chance involving money) within the past 12 months, or at any earlier point in your life?” Only those who responded affirmatively were eligible to proceed with the survey. A total of 674 participants were recruited using purposive sampling, selected to ensure the inclusion of individuals with current or prior experience in online gambling. This strategy aligned with the study’s aim of examining gambling-related behavior and their psychological correlates within a targeted and relevant population (see Table 1 for demographic details).

**Table 1:** Demographic and background characteristics of participants in Study-I

Variable	Category	*N*	%
Gender	Male	422	62.6%
	Female	252	37.4%
Age	M = 35.23, *SD* = 16.30		
Educational attainment	Primary or secondary education	100	14.8%
	High school	156	23.1%
	Undergraduate degree	378	56.1%
	Postgraduate degree	40	5.9%
Marital status	Married	485	72%
	Single	189	28%
Psychological support history (general mental health support, not gambling-specific)	Currently receiving treatment	32	4.7%
	Past treatment	351	52.1%
	Never received treatment	291	43.2%
Emotional influence on daily behavior	Rarely	39	5.8%
	Sometimes	118	17.5%
	Often	223	33.1%
	Very often	294	43.6%
Coping via online gambling or digital behavior	Never	26	3.9%
	Rarely	132	19.6%
	Occasionally	287	42.6%
	Often	229	34%

As part of the scale adaptation process, the following translation and cultural validation procedures were implemented for the OPGBI: The OPGBI was translated and culturally adapted into Turkish following internationally recognized guidelines for cross-cultural scale adaptation (Beaton et al., 2000; Van de Vijver & Tanzer, 2004). Prior to the adaptation process, formal permission to translate and use the scale was obtained from the corresponding author in the original study. The English version of the OPGBI was first translated into Turkish by two bilingual psychologists, one with a doctoral degree in clinical psychology and the other in counseling psychology. An independent expert in English language education, who was blind to the original items, then performed a back-translation into English. Discrepancies between the original and back-translated versions were evaluated and resolved by two additional experts with specialization in clinical/counseling and cross-cultural psychology to ensure both semantic and conceptual equivalence. The finalized Turkish version was subsequently pilot-tested with participants to assess item clarity and comprehensibility. No major modifications were considered necessary following the pilot phase.

Besides these, all participants provided informed consent digitally before accessing the survey. Ethical approval for the study was obtained from the Human Research Ethics Committee (BLINDED FOR REVIEW). Participation was voluntary and anonymous, and participants were informed of their right to withdraw from the study at any point without penalty. No financial or other incentives were provided. All data were collected in accordance with institutional and ethical research guidelines.

#### Measure

*Online Problem Gambling Behavior Index (OPGBI).* Originally, it was developed by Auer et al. (2024) to assess behavioral indicators of problematic online gambling, with an emphasis on actual gambling activities rather than perceived consequences. The scale consists of 12 items, each rated on a 4-point Likert-type scale (0 = Never, 3 = Almost Always), with higher scores reflecting greater engagement in maladaptive online gambling behavior. Exploratory factor analysis (EFA) conducted during the original validation study supported a three-factor structure encompassing: (1) Gambling Behavior (e.g., “*Do you increase your stakes after losing in an online gambling session?*”), (2) Limit-Setting (e.g., “*Do you hit your [or the website’s] money spending limits [if you have any]?*”), and (3) Communication with Operators (e.g., “*Do you contact customer services to complain about your online gambling losses?*”). These three latent dimensions accounted for 46% of the total variance, with the Gambling Behavior factor explaining the largest share (22%; eigenvalue = 2.84). Standardized factor loadings for items meeting the recommended threshold of 0.40 ranged from 0.52 to 0.97, and corrected item-total correlations varied between 0.29 and 0.75. The scale demonstrated high internal consistency (Cronbach’s α = 0.82) and acceptable model fit indices. Additionally, the OPGBI exhibited good convergent validity, explaining 51.8% of the variance in Problem Gambling Severity Index (PGSI) scores.

#### Data Analysis

To evaluate the construct validity, internal consistency, and measurement invariance of the Turkish adaptation of the OPGBI, a series of psychometric analyses were conducted. A CFA was first performed using the maximum likelihood estimation method to test the three-factor structure proposed in the original development study (Auer et al., 2024). This model includes the sub-dimensions of gambling behavior, limit-setting, and communication with operators. CFA was used to confirm the factor structure identified in the original version rather than to explore new latent dimensions.

**Fig. 1:**
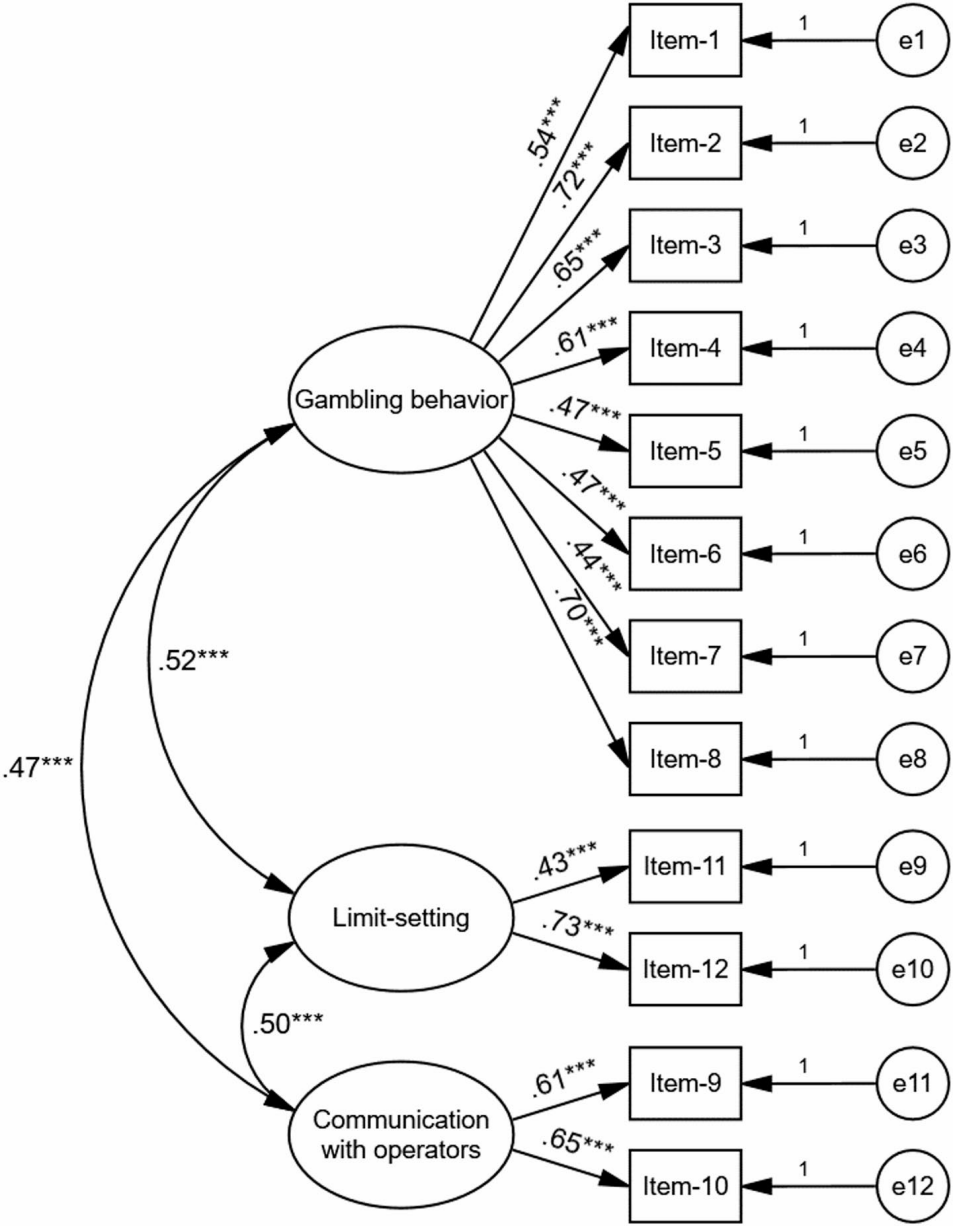
Standardised CFA model of the OPGBI. *Note.* *** *p* <.001

Model fit was evaluated using the Comparative Fit Index (CFI ≥ 0.90), Tucker-Lewis Index (TLI ≥ 0.90), Root Mean Square Error of Approximation (RMSEA ≤ 0.08), and Standardized Root Mean Square Residual (SRMR ≤ 0.08), in line with established cut-offs (Hu & Bentler, 1999). The chi-square statistic (χ²) and degrees of freedom (df) were also reported, with recognition that χ² is sensitive to large sample sizes. Fit indices and standardized factor loadings are presented in Table 2; Fig. 1, respectively. Internal consistency reliability was assessed using Cronbach’s alpha for each subscale and the total score, with α ≥ 0.70 considered acceptable (Nunnally & Bernstein, 1994). In addition, corrected item-total correlations were computed to evaluate item discrimination, with values above 0.30 interpreted as satisfactory (cf. Table 2). To test for measurement invariance across gender, a multi-group CFA was conducted to evaluate configural, metric, and scalar invariance. Model comparisons were examined using changes in CFI (ΔCFI ≤ 0.01) and RMSEA (ΔRMSEA ≤ 0.015), following the recommendations of Cheung and Rensvold (2002). Detailed model fit indices and invariance test results are reported in Table 3.

**Table 2:** Descriptive statistics, corrected item-total correlations, and standardized factor loadings for the OPGBI

Items	M	SD	Corrected item-total correlations	Standardized factor loadings
OPGBI – 1	2.49	0.97	0.62	0.54
OPGBI – 2	2.95	0.89	0.39	0.72
OPGBI – 3	2.13	1.12	0.38	0.65
OPGBI – 4	2.57	1.03	0.38	0.61
OPGBI – 5	2.68	0.90	0.42	0.47
OPGBI – 6	1.92	1.05	0.50	0.47
OPGBI – 7	2.70	0.86	0.47	0.44
OPGBI – 8	2.09	1.21	0.42	0.70
OPGBI – 9	1.94	0.95	0.54	0.61
OPGBI − 10	3.18	1.12	0.36	0.65
OPGBI – 11	3.20	0.97	0.42	0.43
OPGBI – 12	2.98	1.06	0.45	0.73

**Table 3:** Model fit indices and measurement invariance results for the OPGBI across gender

Model	χ²	df	CFI	TLI	RMSEA	SRMR	ΔCFI	ΔRMSEA
Configural invariance	212.45	109	0.934	0.912	0.052	0.049	-	-
Metric invariance	218.67	117	0.931	0.910	0.051	0.052	0.003	0.001
Scalar invariance	229.15	125	0.926	0.905	0.053	0.055	0.005	0.002

To assess common method bias (CMB), Harman’s single-factor test was conducted. All 12 items of the OPGBI were entered into an unrotated EFA using principal axis factoring to determine whether a single factor accounted for the majority of the variance. A single factor accounting for more than 50% of the total variance would suggest the presence of CMB (Podsakoff et al., 2003). Although widely used, Harman’s test is limited in detecting subtle sources of method variance and is considered a preliminary diagnostic rather than a definitive test. However, given that Study-I involved a single self-report instrument measured at one-time point, Harman’s test was deemed appropriate for this phase of the study. All statistical analyses in this process were performed using IBM SPSS (v. 23) for descriptive and reliability analyses and AMOS (v. 23) for CFA and measurement invariance testing.

## Results

To evaluate the factorial structure of the Turkish version of the OPGBI, a CFA was conducted based on the three-dimensional structure proposed by Auer et al. (2024). The model comprising Gambling Behavior, Limit-Setting, and Communication with Operators demonstrated an excellent fit to the data, χ² (102) = 223.71, CFI = 0.937, TLI = 0.925, RMSEA = 0.05, and SRMR = 0.039, all of which met established criteria for adequate model fit (Hu & Bentler, 1999) (cf. Figure 1). All items loaded significantly on their respective latent dimensions (*p* <.001), with standardized factor loadings ranging from 0.43 to 0.73. The Gambling Behavior subscale (Items 1–8) yielded loadings between 0.44 and 0.72, Limit-Setting (Items 11–12) ranged from 0.43 to 0.73, and Communication with Operators (Items 9–10) showed loadings of 0.61 and 0.65. Notably, Item 8 previously flagged in the original development study due to insufficient loading demonstrated a robust factor loading of 0.70 and was retained based on both statistical and conceptual criteria. Inter-factor correlations were moderate and statistically significant, indicating meaningful but distinct sub-dimensions: *r* =.52 (Gambling Behavior ↔ Limit-Setting), *r* =.47 (Gambling Behavior ↔ Communication), and *r* =.50 (Limit-Setting ↔ Communication).

Moreover, multiple indices were employed to evaluate internal consistency. Cronbach’s α values exceeded conventional thresholds for all subscales: 0.84 for Gambling Behavior, 0.76 for Limit-Setting, and 0.81 for Communication with Operators. The total scale exhibited a Cronbach’s α of 0.88, indicating high overall reliability. Complementary estimates further confirmed the scale’s psychometric robustness. McDonald’s omega (ω) values were 0.85, 0.77, and 0.82 for the respective sub-scales and 0.89 for the total scale. Similarly, Guttmann’s λ-6 coefficients were 0.83 (Gambling Behavior), 0.75 (Limit-Setting), 0.80 (Communication), and 0.87 for the full scale mirroring the α and ω findings. Corrected item-total correlations ranged from 0.36 to 0.62, supporting the internal coherence and discriminant contribution of each item (cf. Table 2).

To assess the cross-group validity of the scale, measurement invariance across gender was tested using multi-group CFA. The configural model, which assumes equivalent factor structures, yielded a good fit: χ² (109) = 212.45, CFI = 0.934, RMSEA = 0.052, SRMR = 0.049. Subsequent models imposing metric (equal factor loadings) and scalar (equal intercepts) constraints revealed minimal changes in fit indices, ΔCFI < 0.01 and ΔRMSEA < 0.015, thus supporting full measurement invariance across gender groups (Cheung & Rensvold, 2002). These results indicate that the OPGBI operates equivalently among male and female participants, permitting valid cross-group comparisons (cf. Table 3). Furthermore, given the reliance on self-report measures within a single administration context, Harman’s single-factor test was employed as a diagnostic for potential common method bias (Podsakoff et al., 2003). An unrotated EFA revealed that the first factor accounted for 23.6% of the total variance, substantially below the critical 50% threshold. This result suggests that common method variance was unlikely to have substantially influenced the observed associations. The adapted scale and all items in Turkish are presented in Appendix-A.

### Study-II

While Study-I focused on the psychometric validation of the Turkish version of the OPGBI, Study-II aimed to examine the psychological mechanisms underlying problematic online gambling behavior. Guided by the EDM (Weiss et al., 2012), this phase investigated how emotional reactivity contributes to problematic gambling engagement and whether this relationship is mediated by psychological distress, specifically symptoms of depression, anxiety, and stress.

Emotional reactivity refers to the intensity and duration of individuals’ affective responses to internal or external stimuli. Elevated emotional reactivity has been shown to increase vulnerability to maladaptive coping strategies, including risky or repetitive online behaviors such as gambling (Blaszczynski & Nower, 2002; Gratz & Roemer, 2004). Psychological distress manifesting through depressive, anxious, or stress-related symptomatology has similarly been linked to problematic gambling as a form of affect regulation or experiential avoidance (Blaszczynski & Nower, 2002; Williams et al., 2012). Building on this framework, the present study tested a parallel mediation model in which emotional reactivity was hypothesized to be positively associated with each of the three OPGBI sub-dimensions, with this relationship mediated separately by symptoms of depression, anxiety, and stress.

#### Participants and Procedure

Study-II employed an independent sample from Sample-I to investigate the hypothesized psychological mechanisms underlying problematic online gambling behavior. Data were collected via an online, self-report survey administered through a digital platform. Participants were invited through targeted announcements posted on social media and digital communities related to online gambling (e.g., Telegram betting groups and Twitter/X gambling-related community pages). The recruitment approach mirrored that in Study-I and was designed to reach individuals with relevant gambling experience. A total of 813 individuals were recruited using purposive sampling criteria designed to capture participants with current or previous involvement in digital gambling contexts, thereby maximizing the relevance of psychological constructs under examination. Participation was anonymous and entirely voluntary. All participants provided informed consent before beginning the study (see Table 4 for demographic details).

**Table 4:** Demographic and background characteristics of participants in Study-II

Variable	Category	*N*	%
Gender	Male	528	65%
	Female	285	35%
Age	M = 34.76, *SD* = 11.50		
Educational attainment	Primary or secondary education	36	4.4%
	High school	156	19.2%
	Undergraduate degree	489	60.2%
	Postgraduate degree	132	16.2%
Marital status	Married	544	66.9%
	Single	269	33.1%
Psychological support history (general mental health support, not gambling-specific)	Currently receiving treatment	72	8.9%
	Past treatment	403	49.6%
	Never received treatment	338	41.6%
Emotional influence on daily behavior	Rarely	112	13.8%
	Sometimes	274	33.7%
	Often	102	12.5%
	Very often	325	40%
Coping via online gambling or digital behavior	Never	36	4.4%
	Rarely	197	24.2%
	Occasionally	362	44.5%
	Often	218	26.8%

#### Measures

*Emotional Reactivity Scale (ERS).* originally developed by Nock et al. (2008), the assessment measures individuals’ tendencies to experience heightened emotional responses, particularly in interpersonal contexts and their ability to regulate such states. The Turkish adaptation of the scale was conducted by Seçer et al. (2013). The instrument consists of 21 items rated on a 4-point Likert-type scale (1 = strongly disagree, 4 = strongly agree), with higher scores indicating greater emotional reactivity. The scale comprises three sub-dimensions: emotional sensitivity (e.g., *“I get emotional very easily in the face of upsetting events”*), emotional reactivity (e.g., *“If I have a disagreement with someone*,* it takes me a long time to get over it”*), and psychological resilience (e.g., *“I get hurt emotionally very easily”*). In the Turkish adaptation, the factor loadings were found to range between 0.34 and 0.82, while the Cronbach’s α coefficients for the sub-scales ranged from 0.76 to 0.88, demonstrating satisfactory levels of internal consistency. For this study, only the 7-item emotional reactivity subscale was used, demonstrating high internal consistency (Cronbach’s α = 0.89).

*Depression Anxiety Stress Scale-21 (DASS-21).* is a shortened version of the original 42-item scale developed by Lovibond and Lovibond (1995) to assess symptoms of depression, anxiety, and stress in clinical and non-clinical populations. Each sub-scale as depression (e.g., *“I realized I couldn’t experience any positive emotions”*), anxiety (e.g., *“I felt like I was close to panicking”*), and stress (e.g., *“I felt like I was using up too much of my nervous energy”*) consists of 7 items, rated on a 4-point Likert scale (0 = did not apply to me at all, 3 = applied to me very much or most of the time), with higher scores reflecting greater symptom severity. The Turkish adaptation of the DASS-21 was conducted by Yılmaz et al. (2017), with factor loadings ranging from 0.41 to 0.81 and Cronbach’s α values between 0.76 and 0.82. In the present sample, the scale demonstrated high internal reliability, with Cronbach’s α values of 0.81 for depression, 0.84 for anxiety, and 0.89 for stress.

*Online Problem Gambling Behavior Index (OPGBI).* originally developed by Auer et al. (2024), the OPGBI was designed to assess actual behavioral patterns of problematic online gambling rather than retrospective perceptions of harm. The instrument includes 12 items rated on a 4-point Likert-type scale (1 = never, 4 = almost always), with higher scores indicating a greater frequency of maladaptive gambling behaviors. Items are grouped into three sub-dimensions: Gambling Behavior (e.g., “*Do you increase your stakes after losing in an online gambling session?*”), Limit-Setting (e.g., “*Do you hit your [or the website’s] money spending limits [if you have any]?*”), and Communication with Operators (e.g., “*Do you contact customer services to complain about your online gambling losses?*”). In the original validation, factor loadings ranged from 0.52 to 0.97 and corrected item-total correlations between 0.29 and 0.75. The scale accounted for 46% of total variance, with the Gambling Behavior factor explaining the largest share. In the current study, the OPGBI showed excellent reliability across sub-scales: α = 0.84, 0.76, and 0.81, respectively.

#### Data Analysis

To examine the hypothesized mediation model, structural equation modeling (SEM) was conducted. The analytical framework tested whether emotional reactivity, as measured by the ERS, predicted three dimensions of problematic online gambling behavior, namely gambling behavior, limit-setting, and communication with operators and whether these relationships were mediated by psychological distress, operationalized through the depression, anxiety, and stress subscales of the DASS-21.

A parallel multiple mediation structure was specified in which emotional reactivity served as the independent variable, each DASS subscale was modeled as a distinct mediator, and the three OPGBI sub-dimensions were simultaneously entered as dependent variables. This structure enabled the estimation of direct, indirect, and total effects across all pathways while accounting for shared variance among mediators and outcomes. Model estimation was performed using maximum likelihood estimation (MLE). To evaluate model fit, multiple global indices were employed in accordance with established recommendations (Hu & Bentler, 1999). These included the Comparative Fit Index (CFI), Tucker–Lewis Index (TLI), Root Mean Square Error of Approximation (RMSEA), and the Standardized Root Mean Square Residual (SRMR). Values above 0.90 for CFI and TLI, and below 0.08 for RMSEA and SRMR, were considered indicative of adequate fit. The chi-square statistic (χ²) and associated degrees of freedom (df) were also reported, although interpreted with caution due to its sensitivity to sample size. The significance of indirect effects was tested using a bias-corrected bootstrap procedure with 10,000 resamples and 95% confidence intervals, as recommended by Preacher and Hayes (2008). Indirect effects were interpreted as statistically significant when the confidence intervals (CIs) did not include zero. Bootstrapping was selected for its robustness in estimating indirect effects, particularly in complex mediation models with non-normal sampling distributions.

In contrast to Study-I, which employed Harman’s single-factor test, Study-II adopted more thorough statistical controls for potential CMB, consistent with best practices in multi-construct SEM research. Prior to conducting the SEM, all relevant assumptions were verified, including multivariate normality, absence of multicollinearity, and model identification. Descriptive statistics, correlation matrices, and internal consistency coefficients were calculated using IBM SPSS (v.23), while all SEM procedures and mediation analyses were conducted in AMOS (v.23).

## Results

The SEM demonstrated a satisfactory overall fit to the data, supporting the adequacy of the hypothesized parallel mediation framework. Fit statistics indicated good model fit: χ² (127, *N* = 813) = 324.15, *p* <.001, CFI = 0.932, TLI = 0.918, RMSEA = 0.054, and SRMR = 0.041. All indices met conventional thresholds for acceptable model fit (Hu & Bentler, 1999), suggesting that the data adequately represented the proposed structure.

**Fig. 2:**
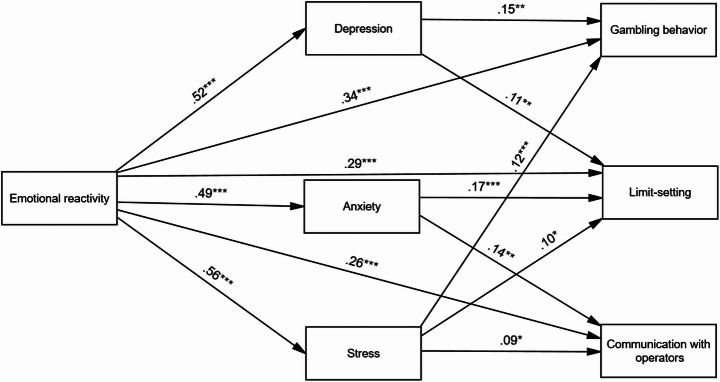
SEM showing direct and indirect effects of emotional reactivity on online problematic gambling behavior through psychological distress *Note.* This figure illustrates a mediation model tested with cross-sectional data and does not imply causal directionality. Pathways are specified based on theoretical assumptions from the EDM. *** *p* <.001, ** *p* <.01, * *p* <.05. All estimates are standardized regression weights

Consistent with H_1_, emotional reactivity was significantly and positively associated with all three dimensions of problematic online gambling behavior. Direct effects were observed from emotional reactivity to gambling behavior (β = 0.34, *p* <.001), limit-setting (β = 0.29, *p* <.001), and communication with operators (β = 0.26, *p* <.001), indicating that individuals with heightened emotional reactivity reported greater engagement in maladaptive online gambling behaviors across all domains. In line with H_2a–2c_, emotional reactivity was also significantly associated with all three psychological distress variables: depression (β = 0.52, *p* <.001), anxiety (β = 0.49, *p* <.001), and stress (β = 0.56, *p* <.001), each of which functioned as a potential mediator in the model. These mediators, in turn, exhibited differential predictive effects on the OPGBI sub-dimensions. Depression significantly predicted gambling behavior (β = 0.15, *p* <.01) and limit-setting (β = 0.11, *p* <.01), though its association with communication was not significant (β = 0.06, *p* =.087). Anxiety was a significant predictor of limit-setting (β = 0.17, *p* <.001) and communication (β = 0.14, *p* <.01), but its effect on gambling behavior did not reach statistical significance (β = 0.08, *p* =.066). Stress emerged as a consistent predictor across all three outcome domains, significantly predicting gambling behavior (β = 0.12, *p* <.001), limit-setting (β = 0.10, *p* <.05), and communication with operators (β = 0.09, *p* <.05) (cf. Figure 2).

Bootstrapping procedures with 10,000 bias-corrected resamples provided additional support for several indirect (mediated) effects. The indirect effect of emotional reactivity on gambling behavior through depression was statistically significant (β = 0.08, 95% CI [0.03, 0.14]), as was the path through stress (β = 0.07, 95% CI [0.02, 0.12]). For limit-setting, significant indirect effects were found via both anxiety (β = 0.08, 95% CI [0.04, 0.13]) and stress (β = 0.06, 95% CI [0.01, 0.11]). Similarly, communication with operators was indirectly influenced by emotional reactivity through anxiety (β = 0.07, 95% CI [0.02, 0.12]) and stress (β = 0.05, 95% CI [0.01, 0.09]). All reported indirect effects were statistically significant, as their 95% CIs did not include zero (cf. Table 5). Taken together, these results provide full empirical support for H_1_, and partial support for H_2a–2c_, confirming the role of emotional reactivity as a direct predictor of problematic online gambling and identifying stress as the most robust mediator across all outcome domains. While all three indicators of psychological distress contributed meaningfully to the mediation process, their predictive strength varied across sub-dimensions, underscoring the multidimensional nature of affective vulnerability in online gambling behavior.

**Table 5:** Emotional reactivity predicts problematic gambling behavior through psychological distress

Paths	β	95% CI	
*Indirect effects*		LL	UL
Emotional reactivity ◊ Depression ◊ Gambling behavior	0.08	0.03	0.14
Emotional reactivity ◊ Stress ◊ Gambling behavior	0.07	0.02	0.12
Emotional reactivity ◊ Anxiety ◊ Limit-setting	0.08	0.04	0.13
Emotional reactivity ◊ Stress ◊ Limit-setting	0.06	0.01	0.11
Emotional reactivity ◊ Anxiety ◊ Communication with operators	0.07	0.02	0.12
Emotional reactivity ◊ Stress ◊ Communication with operators	0.05	0.01	0.09

Note. Bootstrap sample size = 10,000; LL = lower limit; UL = upper limit; CI = confidence interval. All indirect effects are bias-corrected using percentile bootstrapping. Direct paths and mediation values are standardized regression coefficients (β)

## Discussion

This two-phase investigation aimed to both adapt the OPGBI into the Turkish and empirically examine the emotion-regulatory mechanisms contributing to problematic online gambling behavior. Framed within the EDM (Weiss et al., 2012), the study contributes a significant theoretical and empirical contribution to the behavioral addiction literature by integrating psychometric validation with mediation-based modeling of emotional and psychological risk pathways.

In the first study, the psychometric properties of the Turkish OPGBI were examined in detail. The CFA supported a three-factor structure consistent with the original instrument, comprising gambling behavior, limit-setting, and communication with gambling operators, demonstrating good factorial validity, high internal consistency, and gender-based measurement invariance. This not only affirms the structural integrity of the scale in a non-Western context but also positions it as a valuable tool for assessing behavioral indicators of online gambling across diverse cultural settings. Unlike conventional measures that focus on gambling severity or negative consequences, the OPGBI operationalizes discrete behavioral patterns, yielding greater specificity in identifying risky digital gambling behaviors prior to clinical onset. Moreover, the cultural adaptation process responds to the urgent need for psychometric tools that reflect socioculturally embedded behavioral norms, particularly in rapidly digitizing societies such as Türkiye. By ensuring semantic and conceptual equivalence during translation and by empirically validating its structure across gender, the scale’s utility is extended beyond mere linguistic conversion to meaningful cross-cultural application.

Building on this psychometric foundation, Study-II employed a theory-driven SEM to examine the affective pathways that connect emotional reactivity to problematic online gambling behavior. Consistent with H_1_, emotional reactivity emerged as a significant predictor of all three OPGBI dimensions. This finding substantiates the core assertion of the EDM that emotionally reactive individuals are more likely to engage in maladaptive coping behaviors, such as persistent gambling, to manage or suppress distressing emotional states (Szekely & Miu, 2015; Velotti et al., 2021; Weiss et al., 2015). Importantly, this association was not limited to gambling frequency alone but extended to dimensions of behavioral control (limit-setting) and platform engagement (communication with operators), suggesting that emotional reactivity undermines gambling restraint and also the individual’s interpersonal and procedural interactions within gambling environments. It is also important to consider how Türkiye’s legal environment may have influenced the findings, particularly with respect to the ‘communication with operators’ dimension. Since most participants accessed unregulated or illegal gambling platforms, they may have felt reluctant or unsafe to engage directly with operators, fearing data misuse or legal exposure. This could have dampened or distorted their reported communication behavior, regardless of psychological factors, and may partially account for weaker associations observed in this domain. Future studies conducted in legally regulated environments could help disentangle these contextual effects.

Hypotheses 2a through 2c received partial but theoretically compelling support. The mediating roles of depression, anxiety, and stress revealed distinct affective pathways through which emotional reactivity is associated with variations in gambling behavior. Specifically, depression and stress mediated the effects of emotional reactivity on gambling behavior and limit-setting, whereas anxiety predominantly influenced communication and limit-setting but not core gambling behavior. Stress was the only mediator to predict all three dimensions significantly, highlighting its role as a pervasive affective vulnerability. These findings are parallel with transdiagnostic models of behavioral addiction, which emphasize the role of negative affect and impaired emotion regulation as central mechanisms of risk (Estevez et al., 2017; Gonzalez-Roz et al., 2024; Loreto et al., 2024). It is important to clarify that, although the mediation model presented in Fig. 2 was grounded in theoretical assumptions derived from the EDM, it was tested using cross-sectional data. Therefore, the depicted pathways should not be interpreted as evidence of causality. While the model reflects a theoretically informed direction from emotional reactivity to psychological distress and gambling behavior, alternative or bidirectional relationships (e.g., psychological distress influencing emotional reactivity) are equally plausible. Thus, these associations should be interpreted as correlational, pending further longitudinal or experimental validation.

Furthermore, depression’s mediation suggests that low mood and emotional disengagement may lead to gambling as a strategy for numbing or escape, echoing prior evidence that depressive states promote cognitive distortions and persistence in gambling episodes (Alaba- Ekpo et al., 2024; Blaszczynski & Nower, 2002; Daughters et al., 2005). The differentiated role of anxiety impacting control and reactivity but not direct behavior may be interpreted through its physiological correlates, such as somatic arousal and heightened vigilance, which interfere with decision-making and provoke reactive interactions with platforms, especially under perceived loss or uncertainty (Broman-Fulks et al., 2014). Most notably, the consistent mediation effects of stress align with conceptualizations of chronic emotional overload as a generalized driver of maladaptive coping. Stress, as opposed to episodic mood disturbances, appears to exert broad impairing effects on self-regulation, leading to persistent engagement, loss of boundary-setting, and emotionally reactive communication (Flores-Kanter et al., 2021; Kim et al., 2013).

Together, these findings suggest a detailed extension of the EDM. Rather than conceptualizing emotional reactivity as leading to gambling behavior via a uniform mechanism of “distress,” the present model shows that discrete affective states depression, anxiety, and stress differentially mediate the pathway depending on the specific behavioral dimension. In this way, the results affirm the EDM’s general framework while enriching its specificity: emotional dysregulation is not a monolithic vulnerability but one that operates through varying symptom clusters with domain-specific behavioral consequences. These distinctions both clarify inconsistencies in previous (national) literature and support calls to break down the umbrella term ‘problem gambling’ into functional, theory-informed subdomains.

From a clinical perspective, the study can suggest that interventions aimed at gambling behavior can attend more closely to the emotional reactivity profile of individuals and the dominant distress mechanisms driving their behavioral engagement. Emotion-focused treatments, such as Dialectical Behavior Therapy (DBT), transdiagnostic CBT, or mindfulness-based emotion regulation training, may hold particular value for individuals exhibiting elevated emotional sensitivity and reactivity. Moreover, stress emerged as the most consistent and generalizable mediator, suggesting that screening for stress-related symptoms or chronic affective strain could act as a useful clinical marker for gambling vulnerability. These implications share common ground with the emphasis in addiction studies on affect regulation and transdiagnostic risk markers, moving beyond surface-level behaviors to address the emotional scaffolding that sustains them (Estevez et al., 2023; Rogier & Velotti, 2018; Thurm et al., 2023; Velotti et al., 2021). All things considered, the present research provides a comprehensive account of how emotion regulation deficits and psychological distress are linked with patterns of problematic gambling behavior in online contexts. By combining meticulous psychometric validation with theory-based mediation modeling, the study contributes both a reliable tool and an explanatory framework for understanding online gambling risk. Through this, we can say that it not only strengthens the conception centrality of emotional dysregulation in behavioral addictions but also opens the door to more targeted, impact-focused interventions in emerging gambling cultures.

### Limitations and Implications

Despite the theoretical and empirical contributions of the present study, several limitations can be acknowledged. First, the cross-sectional design precludes any definitive conclusions regarding causality. Although the mediation pathways proposed were theoretically grounded and statistically supported, the temporal ordering of variables remains hypothetical. Longitudinal or experimental research would be necessary to determine whether emotional reactivity and psychological distress precede increases in maladaptive online gambling behavior. Second, data were collected exclusively via self-report instruments, which may introduce shared method variance and raise concerns about social desirability or recall biases. These limitations are particularly salient in the Turkish context, where gambling is heavily restricted, and its legal status remains ambiguous. In Türkiye, all forms of casino gambling are officially prohibited, leading to the proliferation of unregulated or illegal online betting platforms. This legal vacuum contributes to the emergence of gambling behaviors that differ from traditional Western gambling patterns and may introduce unique risk factors not accounted for in conventional frameworks. Moreover, the fragmented nature of Turkish gambling legislation spread across multiple legal texts creates a sociopolitical environment in which gambling occupies a legally and morally contested space (Erkin, 2022). As a result, individuals may underreport their gambling involvement due to fear of legal consequences or social stigma, thereby affecting the accuracy of self-reported data and potentially limiting participation in gambling-related studies. Third, although both samples were sizeable and demographically diverse, they were drawn using purposive sampling from online populations with current or past gambling experience, which may limit the generalizability of findings to broader populations, including adolescents or non-internet users. Fourth, the study did not differentiate among types of online gambling platforms (e.g., sports betting, slot machines, online casino games, etc.), which may be associated with distinct psychological and behavioral profiles. Future research can assess platform-specific engagement to better contextualize problematic gambling patterns. Ultimately, the study focused specifically on emotional reactivity and psychological distress, excluding other potentially influential variables such as impulsivity, trauma history, or cognitive distortions, which can be examined in future research to enrich the explanatory model.

Moreover, the findings of this study provide both theoretically grounded and practically actionable implications across clinical, research, and public health domains. Most notably, the results underscore the centrality of emotion regulation difficulties, particularly heightened emotional reactivity and chronic psychological distress, as key transdiagnostic mechanisms underlying problematic online gambling. The consistent mediating role of stress across all behavioral dimensions suggests that chronic affective strain may operate as a general vulnerability factor. Accordingly, the early identification of emotionally reactive individuals, particularly those experiencing persistent stress, may indicate a viable pathway for preventative intervention before gambling behavior becomes clinically impairing. Therapeutic frameworks such as DBT, mindfulness-based emotion regulation training, and transdiagnostic approaches targeting distress tolerance and affective instability are particularly well-suited to addressing these mechanisms (Aguirre et al., 2024; Schmidt et al., 2024). Such interventions can both reduce gambling frequency and improve behavioral regulation and consequently reduce emotionally reactive participation in gambling platforms. From an assessment standpoint, the Turkish adaptation of the OPGBI provides a psychometrically robust, culturally relevant, and behaviorally specific instrument for evaluating multidimensional patterns of gambling engagement. Its structure can allow clinicians and researchers to assess discrete behavioral tendencies, including excessive play, failed limit-setting, and maladaptive communication with operators, each of which may reflect distinct emotional antecedents and require differentiated clinical responses. The scale’s applicability is considered to extend to use in early risk monitoring, digital mental health platforms, and individualized treatment planning. Besides, it delivers a valuable tool for identifying gambling risk in non-Western populations, filling a notable gap in the cross-cultural gambling literature. At the level of public health and policy, the findings call for a paradigm shift in the conceptualization and prevention of gambling-related harm. Existing frameworks often focus on financial loss or gambling frequency as primary indicators of risk, neglecting the emotional and psychological processes that drive such behaviors.

The present study highlights the need for prevention strategies that directly address affective dysregulation, particularly in sociocultural contexts where gambling is simultaneously stigmatized, under-regulated, and concealed. In Türkiye, where formal casino gambling is illegal and online gambling proliferates through unregulated platforms, individuals with heightened emotional vulnerability are exposed to significant risk in the absence of institutional safeguards. Furthermore, the fragmented nature of Turkish gambling legislation and the lack of coordinated public discourse contribute to a socio-legal climate in which help-seeking is suppressed and behaviors are hidden. In this context, the integration of emotion-focused education, harm reduction, and culturally tailored screening tools becomes especially urgent. National prevention campaigns, public health initiatives, and policy reform can be aligned with these psychological dimensions to ensure that gambling regulation does not merely target financial consequences but also addresses the emotional needs that sustain behavioral addiction. To summarize, this research underscores the importance of embedding emotional and psychological factors into both clinical intervention and public policy. By identifying emotional reactivity and psychological distress as key drivers of online gambling behavior, and by validating a culturally grounded assessment tool, the present study contributes both to the academic understanding of gambling etiology and to the practical task of designing more responsive, affect-informed, and culturally appropriate prevention and intervention efforts.

## Conclusion

By uniting psychometric precision with theoretical depth, this two-phase study yields a comprehensive account of how emotional vulnerability contributes to problematic online gambling behavior within a culturally distinct setting. The validation of the Turkish OPGBI not only extends the reach of behavioral assessment tools into underrepresented populations but also affirms the instrument’s multidimensional structure as a meaningful framework for capturing diverse patterns of digital gambling engagement. More importantly, the structural model built upon the EDM suggests that emotional reactivity is associated with problematic gambling through depression, anxiety, and stress, each of which relates to gambling behavior in psychologically distinct ways. This disaggregation of affective pathways marks an important shift in how emotional mechanisms are conceptualized in the etiology of behavioral addictions.

Situated within the sociocultural and legal dynamics and challenges of Türkiye’s gambling landscape, the findings underscore the urgency of designing emotion-focused interventions and culturally sensitive risk-monitoring systems for populations exposed to unregulated digital gambling environments. As gambling behavior increasingly unfolds in spaces beyond regulatory oversight and psychological visibility, the need to understand its emotional architecture becomes not only clinically relevant but ethically imperative as well. All things considered, this study moves the field toward a more detailed, affect-informed, and globally attuned understanding of gambling behavior, one that recognizes emotional vulnerability not merely as a correlate of risk but as its psychological engine.

## Data Availability

The data from the current study are available from the corresponding author upon reasonable request.

## Appendix-A

### Açıklama

Çevrimiçi Problemli Kumar Davranışı İndeksi (ÇPKDİ) bireylerin son bir ay içerisindeki çevrimiçi kumar davranışlarının sıklığını değerlendiren 12 maddeden oluşmaktadır. Her bir madde, 0 = Hiçbir zaman, 1 = Bazen, 2 = Çoğu zaman ve 3 = Neredeyse her zaman olacak şekilde 4’lü Likert tipi bir derecelendirme ölçeğiyle yanıtlanmaktadır. Ölçeğin alt boyutları ve ilgili maddeler şu şekildedir.

Çevrimiçi Kumar Davranışı: Madde 1, 2, 3, 4, 5, 6, 7 ve 8, Limit Belirleme: Madde 11 ve 12, Operatörle İletişim: Madde 9 ve 10.

Ölçekten alınabilecek toplam puan 0 ile 36 arasında değişmekte olup, yüksek puanlar bireyin daha problemli çevrimiçi kumar davranışları sergilediğine işaret etmektedir.

**Table:**

Maddeler	Hiçbir zaman	Bazen	Çoğu zaman	Neredeyse her zaman
**1.**Çevrimiçi kumar oturumları sırasında hesabınızı tekrar doldurur musunuz?	0	1	2	3
**2.**Çevrimiçi bir kumar oturumunda kaybettikten sonra bahislerinizi artırır mısınız?	0	1	2	3
**3.**Çevrimiçi bir kumar oturumunda kaybettikten sonraki gün bahislerinizi artırır mısınız?	0	1	2	3
**4.**Günde 4 saatten daha uzun süre çevrimiçi kumar oynar mısınız?	0	1	2	3
**5.**Farklı miktarlarda bahislerle çevrimiçi kumar oynar mısınız?	0	1	2	3
**6.**Bir ay içinde beşten fazla çevrimiçi kumar oyunu oynar mısınız?	0	1	2	3
**7.**Kazandıktan hemen sonra tekrar kumar oynar mısınız?	0	1	2	3
**8** Bir çevrimiçi kumar oturumu sırasında hesabınızı doldurmak için birden fazla banka veya kredi kartı kullanır mısınız?	0	1	2	3
**9.**Çevrimiçi kumar sohbet odalarında agresif davranır mısınız?	0	1	2	3
**10.**Çevrimiçi kumar kayıplarınız hakkında şikayette bulunmak için müşteri hizmetlerine başvuruyor musunuz?	0	1	2	3
**11.**Kendi (veya sitenin) para harcama limitlerine ulaşıyor musunuz (varsa)?	0	1	2	3
**12.**Kendi (veya sitenin) zaman harcama limitlerinize (eğer varsa) ulaşır mısınız?	0	1	2	3
